# The Implementation of FDG PET/CT for Staging Bladder Cancer: Changes in the Detection and Characteristics of Occult Nodal Metastases at Upfront Radical Cystectomy?

**DOI:** 10.3390/jcm12103367

**Published:** 2023-05-09

**Authors:** Sarah M. H. Einerhand, Lotte G. Zuur, Maurits J. Wondergem, Thierry N. Boellaard, Kurdo Barwari, Pim J. van Leeuwen, Bas W. G. van Rhijn, Laura S. Mertens

**Affiliations:** 1 Department of Surgical Oncology (Urology), Netherlands Cancer Institute, 1066 CX Amsterdam, The Netherlands; 2 Department of Nuclear Medicine, Netherlands Cancer Institute, 1066 CX Amsterdam, The Netherlands; 3 Department of Radiology, Netherlands Cancer Institute, 1066 CX Amsterdam, The Netherlands; 4 Department of Urology, Caritas St Josef Medical Centre, University of Regensburg, 93053 Regensburg, Germany

**Keywords:** bladder cancer, urothelial carcinoma, imaging, staging, CT, FDG PET/CT, radical cystectomy, neoadjuvant chemotherapy, lymph node, metastasis

## Abstract

Occult lymph node (LN)-metastases are frequently found after upfront radical cystectomy (uRC) for bladder cancer (BC). We evaluated whether the implementation of 18F-fluoro-2-deoxy-D-glucose positron emission tomography/computed tomography (FDG PET/CT) influenced nodal staging at uRC. All consecutive BC patients who underwent uRC with bilateral pelvic lymph node dissection (PLND) were identified and divided into two cohorts: cohort A consisted of patients staged with FDG PET/CT and contrast-enhanced CT (CE-CT) (2016–2021); cohort B consisted of patients staged with CE-CT only (2006–2011). The diagnostic performance of FDG PET/CT was assessed and compared with that of CE-CT. Thereafter, we calculated the occult LN metastases proportions for both cohorts. In total, 523 patients were identified (cohort A n = 237, and cohort B n = 286). Sensitivity, specificity, PPV and NPV of FDG PET/CT for detecting LN metastases were 23%, 92%, 42%, and 83%, respectively, versus 15%, 93%, 33%, 81%, respectively, for CE-CT. Occult LN metastases were found in 17% of cohort A (95% confidence interval (CI) 12.2–22.8) and 22% of cohort B (95% CI 16.9–27.1). The median size of LN metastases was 4 mm in cohort A versus 13 mm in cohort B. After introduction of FDG PET/CT, fewer and smaller occult LN metastases were present after uRC. Nevertheless, up to one-fifth of occult (micro-)metastases were still missed.

## 1. Introduction

Standard treatment of non-metastatic muscle-invasive urothelial bladder cancer (MIBC) consists of radical cystectomy (RC) with bilateral pelvic lymph node dissection (PLND) preceded by cisplatin-based chemotherapy [1]. However, approximately half of MIBC patients are cisplatin-ineligible [2]. At the same time, the benefit of neoadjuvant chemotherapy (NAC) is especially evident in locally advanced disease (cT3-4aN0M0), but not in cT2N0-disease [3]. Consequently, a significant proportion of patients are still treated with upfront RC and PLND.

Nodal involvement in the PLND specimen is associated with poor prognosis [4,5]. Despite pre-treatment staging with contrast-enhanced CT (CE-CT), 20 to 40% of patients with clinically node-negative (cN0) MIBC have lymph node (LN) metastases in the PLND specimen (i.e., occult LN metastases) [6]. This indicates that conventional preoperative imaging with CE-CT is not accurate enough. FDG PET/CT is increasingly being used for LN staging before RC. However, investigating its definitive value has proved challenging [7] because histopathology is only reliable without preoperative systemic therapy, and non-pre-treated patients inevitably are a less representative and lower-risk group. Given previous retrospective results [6,8,9,10], additional FDG PET/CT findings are almost impossible to ignore. Furthermore, some studies have chosen qualitative assessment by visual analysis of FDG PET/CT rather than semi-quantitative measurement of standardized uptake values (SUV) [11,12,13,14]. Finally, other studies have used unconventional size metrics such as long-axis measurements [15,16].

To overcome these challenges, the aim of the present study was to evaluate the effect of the implementation of FDG PET/CT as part of routine staging by assessing its diagnostic accuracy for detecting LN metastases and by comparing the rate of occult LN metastases before and after the implementation of FDG PET/CT in two upfront RC-cohorts treated at the same high-volume hospital.

## 2. Materials and Methods

### 2.1. Patients

Using our prospectively-maintained database, we retrospectively identified all consecutive patients who underwent upfront RC with bilateral PLND or staging PLND for MIBC or (very) high-risk T1-bladder cancer (BC) with curative intent at our tertiary referral hospital between 2016 and 2021. All patients in this cohort were staged with CE-CT and FDG PET/CT before definitive treatment. This cohort will be referred to as cohort A. Additionally, we identified consecutive patients who underwent upfront RC with PLND for MIBC or (very) high-risk T1-BC between 2006 and 2011. The patients in this cohort were staged with CE-CT only. This cohort will be referred to as cohort B.

Patients were excluded because (i) they did not undergo a PLND, (ii) they were insufficiently staged, (iii) they were pre-treated with radiotherapy, or (iv) they were pre-treated with chemotherapy.

The period from 2012 to 2015 was a transition period, during which FDG PET/CT was used sporadically, not routinely, and not by everyone. Because of this heterogeneity in use, we did not analyse this period and chose a full FDG PET/CT cohort (cohort A) vs. a full non-FDG-PET/CT cohort (cohort B).

### 2.2. Pretreatment Staging and Evaluation

All patients were clinically staged by physical examination, cystoscopy, transurethral resection (TUR) and imaging. Patients in cohort A underwent staging with both CE-CT imaging of the pelvis, abdomen and chest, and whole-body FDG PET/CT. Patients in cohort B underwent staging with CE-CT only.

CE-CT scans were performed and evaluated as part of routine clinical practice. A determination of cN0 was defined as no clinical evidence of LN metastases on the CE-CT (short axis diameter >1.0 cm).

FDG PET/CT scans were performed and evaluated as part of routine clinical practice as well. FDG PET/CT has been implemented as part of routine staging in our hospital in an effort to reduce the rate of occult nodal metastases in patients who have undergone upfront RC and, consequently, reduce undertreatment, as pathological lymph node status is associated with a poor prognosis [17].

The preparations of the FDG PET/CT followed our hospital’s standard protocol. Patients fasted for six hours before the FDG PET/CT scan. They received oral pre-hydration fluid before an intravenous injection of 190–240 MBq FDG. Subsequently, images were obtained one hour after injection of FDG, and ideally also four to six hours after injection with FDG. The delayed images were obtained to minimize interference of urinary FDG, i.e., to improve the interpretation of the bladder tumour. Experienced nuclear medicine physicians interpreted the FDG PET/CT scan results. FDG PET/CT images were stored on integrated FDG PET/CT scanners (Gemini TF or Gemini TF Big Bore, Philips, Amsterdam The Netherlands). A low-dose CT-scan from the groins to the skull was performed first, directly followed by the PET-scan. Images were corrected for attenuation and anatomical correlation using the CT images. Referred patients occasionally underwent imaging elsewhere. These images were always reviewed by the nuclear medicine physician in our hospital.

All patients were discussed in multidisciplinary rounds. Here, the clinical TNM stage and subsequent treatment were determined, based on all available information [1]. In this analysis, all patients were chemotherapy-naïve, i.e., did not receive preoperative treatment before RC, either because—according to institutional protocol—there was no indication for it, or they were cisplatin-ineligible. Patients were generally considered for neoadjuvant chemotherapy in case of cT3-4aN0M0 disease, and/or lymph-angio-invasion, and/or hydronephrosis, and/or a palpable mass [3]. Patients were considered cisplatin-ineligible when they met at least one of the following criteria: poor performance status (ECOG performance score 2 or higher), poor renal function (GFR <50–60 mL/min), heart failure (NYHA-class 3 or 4), severe neuropathy (grade 2 or higher), or hearing loss [18].

### 2.3. Surgical PLND-Template and Histopathology

The surgical template of PLND has remained the same during the study period. RC included a PLND according to an anatomically clearly defined template, which consisted of removal of the LNs in the region between the genitofemoral nerve, the obturator fossa, and along the internal iliac artery and the common iliac artery, at least up to the crossing of the ureter [19]. Surgery was performed by five experienced staff urologists, using either a robot-assisted or an open approach.

PLND specimens, containing a minimum of two packages per side [20], were sent to the Pathology Department. The specimens were handled by the Pathology Department according to institutional guidelines. The number of resected LNs, diameter of resected LN and the presence of LN metastases were routinely determined. Patients were classified as pathologically node-negative (pN0) or pathologically node-positive (pN+). The size of the positive lymph nodes were described in the histopathology reports and used for analysis.

### 2.4. Statistical Analysis

In cohort A, we first assessed the diagnostic accuracy of CE-CT and FDG PET/CT for the detection of LN metastases. The results (cN0 vs. cN+) of each imaging modality were compared with histology of the PLND specimen (pN0 vs. pN+) on a per-patient basis. Receiver operating characteristic (ROC) curves, area under the curve of the ROC, sensitivity, specificity, positive predictive value (PPV) and negative predictive value (NPV) were calculated [21].

For each imaging modality, we compared overall survival (OS) between cN0 vs. cN+ patients, using the Kaplan-Meier method. OS was defined as the time between surgery and (any) cause of death. If patients were still alive at the end of follow-up, they were censored on that date.

Then, we calculated the occult LN metastases rate (i.e., the number of pN+ patients/the number of cN0 patients). Occult LN metastases are defined as LN metastases at the PLND specimen (pN+) in clinically node-negative (cN0) patients. The cN0 patients were separated from both cohorts and used to perform the calculation. We compared the rate of occult LN metastases at RC between cohort A and cohort B, i.e., after and before the implementation of FDG PET/CT. The occult LN metastases rate and 95% confidence intervals (CIs) in these cohorts were calculated using binomial tests. Inter-cohort differences in occult LN metastases rates were considered statistically significant if the 95% CIs did not overlap.

Data analysis and collection was performed using SPSS version 27.0 for Windows. Descriptive statistics were used to describe the patient and tumour characteristics of our study population.

## 3. Results

### 3.1. Patient Selection and Characteristics

**Figure 1:**
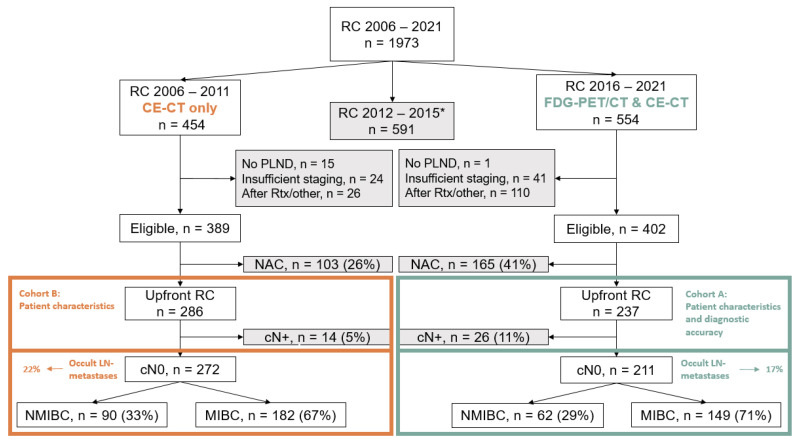
Selection of patients for cohort A and cohort B. Abbreviations: RC = radical cystectomy; CE-CT = contrast enhanced computed tomography; FDG PET/CT = 18F-fluoro-2-deoxy-D-glucose-positron emission tomography with computed tomography; PLND = pelvic lymph node dissection; Rtx = radiotherapy; NAC = neoadjuvant chemotherapy; cN+ = clinically node positive; LN = lymph node; NMIBC = non-muscle invasive bladder cancer; MIBC = muscle invasive bladder cancer; Occult LN metastases = pathologically node-positive patients who were clinically node-negative. * The period from 2012 to 2015 was a transition period, during which FDG PET/CT was not used routinely. Because of this heterogeneity in use, we did not analyse this period and chose a full FDG PET/CT cohort (cohort A) vs. a full non-FDG PET/CT cohort (cohort B).

The selection of patients is shown in Figure 1. A total of 1973 patients underwent RC for BC at our hospital between 2006 and 2021. Patients treated during the “transition period” between 2012 and 2015 were not included in this analysis. A larger percentage of patients were excluded from analysis due to pre-treatment with chemotherapy in cohort A (41%) compared to cohort B (26%). In cohort A, 237 patients remained eligible for analysis. Of these, 211 were staged cN0 as decided during multidisciplinary meetings. In cohort B, 286 patients remained eligible for analysis, of whom 272 were staged cN0.

Clinical patient characteristics of cohort A vs. cohort B are displayed in Table 1. The median age of patients in cohort A was 67 years, as opposed to 63 years in cohort B. The percentage of patients who underwent upfront RC because of diagnosed MIBC was similar in both cohorts.

### 3.2. Diagnostic Accuracy Cohort A

FDG PET/CT was performed in cohort A, so diagnostic accuracy estimates were calculated only for this group. In the 237 patients in cohort A, 47 patients (20%) were pN+ at PLND. Of these, 15% (7 patients) were staged cN+, while 85% (40 patients) were staged as cN0. Sensitivity, specificity, PPV and NPV of FDG PET/CT for detecting LN metastases were 23%, 92%, 42%, and 83%, respectively, compared to 15%, 93%, 33%, and 81%, respectively, for CE-CT, on a per-patient basis. Diagnostic accuracy estimates with corresponding 95% confidence intervals (CI’s) are shown in Table 2.

### 3.3. Occult Lymph Node Metastases

For analysis of occult LN metastases, we focused on the 211 cN0 patients in cohort A and 272 cN0 patients in cohort B.

Of the 211 cN0-patients in cohort A, 36 (17%, 95%; CI 12.2–22.8) had occult metastases (pN+) at the PLND specimen. Among the subgroup of 149 patients with cN0-MIBC, the occult LN metastases rate was 31/149 (21%, 95%; CI 14.6–28.2). The median size of occult LN metastases in cohort A was 4 mm (interquartile range (IQR) 2–10). The histopathological details are shown in Table 3.

For cohort B, 272 cN0 patients were eligible for analysis (Figure 1). Baseline patient and tumour characteristics did not significantly differ from patients in cohort A. Occult LN metastases were found in 59/272 patients (22%, 95%; CI 16.9–27.1). Among the subgroup of 213 patients with cN0-MIBC, the occult LN metastases rate was 55/213 (26%, 95%; CI 20.1–32.2). The median size of occult LN metastases in cohort B was 13 mm (IQR 10–25). Although the occult LN metastases rate was higher than in cohort A, this difference was not statistically significant.

## 4. Discussion

The present study was performed to evaluate whether implementation of FDG PET/CT improved pre-treatment LN staging of patients with MIBC or (very) high risk NMIBC. One of the main findings of this study is that fewer occult LN metastases were found in the PLND specimens after implementation of FDG PET/CT as part of routine staging before RC. Although this difference was not statistically significant, the median size of the missed lymph nodes had substantially decreased (from 13 mm to 4 mm). This finding supports our hypothesis that FDG PET/CT combined with CE-CT is able to detect more and smaller lymph node metastases in anatomically non-enlarged lymph nodes than CE-CT alone. The second finding is that, despite staging with both FDG PET/CT and CE-CT, 17% to 21% of patients still had occult LN metastases at upfront RC. These results illustrate that the “PET” signal emitted by smaller (micro)-metastases often is not sufficient for detection.

Furthermore, we found that the sensitivity of FDG PET/CT for the detection of LN metastases was somewhat higher than that for CE-CT, given comparable specificity. We only included chemo-naive patients who underwent upfront RC because we aimed to compare imaging results with histopathology at PLND; the use of preoperative chemotherapy affects pathological outcomes and is therefore not a reliable gold standard for establishing the accuracy of imaging. Excluding patients treated with preoperative chemotherapy inevitably means that our cohort consisted largely of patients staged as cN0, i.e., lower-risk patients for whom preoperative chemotherapy would not be indicated. Given the selection of patients, the occult LN metastases rate and the negative predictive value are the most reliable “diagnostic parameters” in our cohort. There are several smaller reports (n = 52–93) in which patients treated with NAC were similarly excluded. In these studies, the sensitivity of FDG PET/CT for the detection of LN metastases ranged from 23–64% [11,13,15,22,23]. Negative predictive values reported in these pioneer prospective studies range between 75–91%, consistent with the 83% negative predictive value we report [24,25]. Dason et al. evaluated FDG PET/CT in clinically node-negative patients on CE-CT in a recent retrospective analysis [26]; they found that sensitivity of FDG PET/CT for detection on LN metastases was higher compared to CE-CT, but still low in absolute numbers (23% in patients that were not pre-treated with NAC). Furthermore, they also found that occult LN metastases were usually micro-metastatic (median size 5 mm). Our findings are in line with those reported in the literature and further indicate that—although FDG PET/CT generally detects more and smaller LN metastases than CE-CT—FDG PET/CT also cannot reliably exclude micro-metastatic involvement.

Although differences in diagnostic accuracy between FDG PET/CT with CE-CT and CE-CT alone were not statistically significantly different, FDG PET/CT might still be useful for guiding patient management. FDG PET/CT was immediately used to guide patient management after implementation in our centre. In our study, substantially more patients were excluded from cohort A—i.e., after implementation of FDG PET/CT—because they had received NAC (41%), as compared to cohort B (26%). This difference was present despite the fact that the indications for preoperative chemotherapy in our hospital remained unchanged. This finding suggests an improved selection of patients for preoperative chemotherapy or upfront RC [6,10]. The fact that patients in cohort B also had fewer and smaller (occult) LN metastases in the PLND specimen also indicates improved patient selection after implementation of FDG PET/CT as part of routine pre-treatment staging. Although it cannot be dismissed entirely, the increase in patients receiving NAC is unlikely to be explained by a different case-mix consisting of patients with more advanced disease after implementation of FDG PET/CT, because the 10-year prevalence of patients with MIBC in the Netherlands has remained largely unchanged between 2006 and 2021 [27].

Early stage of microscopic LN metastases often cannot be identified by PET or PET/CT [28]; small lesions may show only minimal uptake of 18F-FDG below the threshold for visualization by the FDG PET/CT. Hence, negative FDG PET/CT findings cannot exclude the presence of a small tumour or a microscopic nodal involvement. At the same time, false-positive findings can occur due to 18F-FDG uptake in infectious or inflammatory tissue (e.g., after transurethral resection) [29]. Another pitfall of FDG PET/CT in bladder cancer imaging is the physiological excretion of FDG, the signal of which can be interpreted as the signal of tumours in the urinary tract. As of now, it remains to be investigated whether this also affects visualization of LN metastases in the surrounding areas in the pelvis.

Apart from considerations such as diagnostic accuracy, implementation of FDG PET/CT will also be determined by its cost-effectiveness and risk-benefit ratio. To date, a comprehensive cost-benefit analysis of the use of an FDG PET/CT for urothelial carcinoma has not been performed. Although FDG PET/CT did detect more and smaller LNs in our study, occult nodal metastases were still present in up to one-fifth of PLND specimens. Hence, our study would warrant critical evaluation of its cost-effectiveness. The prognosis of patients might be determined by the MIBC rather than by the single exposure to radiation from FDG PET/CT. There is risk of false-positive FDG uptake elsewhere. However, a recent report has shown that these findings only lead to clinically significant change in patient management in 1.4% of patients [10]. Therefore, the risk-benefit ratio for implementation of FDG PET/CT might not be unfavourable.

A strength of this study lies in the fact that this is the first to directly compare a RC cohort before and after routine addition of FDG PET/CT to CE-CT. This allowed us to perform both intra-patient and inter-patient comparisons of the imaging modalities. Moreover, this is one of the largest series of patients in the literature with FDG PET/CT in a high-volume tertiary bladder cancer centre, who follow a standardised diagnostic pathway in a specialized BC clinic.

Limitations of this study include those inherent in its retrospective design. Moreover, patients were diagnosed and treated over a relatively long period of time. Both factors make the study susceptible to bias due to interobserver variability in FDG PET/CT and CE-CT assessment and, ultimately, to variation among members of the multidisciplinary team. Imaging and pathology were not centrally reviewed and radiologists and nuclear medicine specialists were not blinded as to other diagnostic information at the time of review. Furthermore, we focused on a subgroup of patients who underwent upfront RC, based on all available diagnostic information, that is, a selected group with relatively low risk of disease. Although we acknowledge selection bias in this cohort, we would also argue that limiting our analysis to patients with upfront RC and PLND produces the most reliable PLND specimen, and that more accurate characterization of nodal status is, clinically, most relevant in these patients as patients with larger (cT3-4) tumours would qualify for preoperative chemotherapy based on this high-risk feature alone.

## 5. Conclusions

Despite these limitations, our results show that, after the introduction of FDG PET/CT for pre-treatment staging of patients with MIBC, relatively fewer and smaller occult LN metastases were found at upfront RC, compared with the time frame when only CE-CT was used. However, up to one-fifth of patients with negative FDG PET/CT still had occult LN metastases. Hence, FDG PET/CT does not replace CE-CT but rather has complementary value in nodal staging of MIBC. Moreover, a negative FDG PET/CT should not be a decisive factor in refraining from neoadjuvant chemotherapy and/or PLND.

## Figures and Tables

**Table 1:** Clinical patient and tumour characteristics in cohort A vs. cohort B.

		Cohort A (n = 237)	Cohort B (n = 286)	*p*-Value
Age, years, mean (SD)		67 (11)	63 (9)	**<0.001**
Sex, n (%)				0.65
	Male	174 (73)	215 (75)	
	Female	63 (27)	71 (25)	
cT-stage, n (%)				0.16
	T1	75 (32)	90 (31)	
	T2	106 (45)	137 (48)	
	T3	48 (20)	41 (14)	
	T4	8 (3)	18 (6)	
cN-stage, n (%)				**0.009**
	N0	211 (91)	272 (95)	
	N+ (CE-CE)	21 (9)	14 (5)	
	N+ (FDG PET/CT + CE-CT)	26 (10)	NA	

Abbreviations: SD = standard deviation; cT-stage = clinical tumour stage, as decided during multidisciplinary rounds; cN-stage, clinically lymph node stage, as decided during multidisciplinary rounds; CE-CT = contrast enhanced computed tomography; FDG PET/CT = 18F-fluoro-2-deoxy-D-glucose-positron emission tomography with computed tomography. These two values are bold because they are statistically significant.

**Table 2:** Diagnostic accuracy of FDG PET/CT versus CE-CT for cohort A.

	FDG PET/CT		CE-CT		
	%	95% CI	%	95% CI	*p*-Value
Sensitivity	23	0.13–0.38	15	0.07–0.29	0.29
Specificity	92	0.87–0.95	93	0.88–0.96	1
Positive predictive value	42	0.24–0.63	33	0.16–0.57	
Negative predictive value	83	0.77–0.88	81	0.76–0.86	
Accuracy	79	0.74–0.84	77	0.72–0.83	
Area under the ROC curve	0.578	0.48–0.68	0.538	0.44–0.63	0.21

Abbreviations: CE-CT = contrast enhanced computed tomography; FDG PET/CT = 18F-fluoro-2-deoxy-D-glucose-positron emission tomography with computed tomography.

**Table 3:** Histopathology of the radical cystectomy and pelvic lymph node dissection specimens of cN0 patients.

		Cohort A (n = 211)	Cohort B (n = 272)	*p*-Value
pT-status (n, %)				0.97
	0	34 (16)	46 (17)	
	Cis/1	63 (30)	81 (30)	
	2	41 (19)	56 (21)	
	3	44 (21)	64 (24)	
	4	16 (8)	25 (9)	
	X	13 (6)	0 (0)	
pN-status (n, %)				0.606
	0	175 (83)	213 (78)	
	1	20 (10)	30 (11)	
	2–3	26 (8)	29 (11)	
Number of lymph nodes removed (median, IQR)		20 (15–25)	15 (12–21)	**<0.001**
Size positive lymph nodes, mm (median, IQR)		4 (2–10)	13 (10–25)	**<0.001**

Abbreviations: pN-status = pathological nodal status; pT-status = pathological tumour stage; IQR = inter quartile range, X = patients that underwent staging PLND and therefore, pathological tumour stage is unknown.

## Data Availability

The data that support the findings of our study are available from the corresponding author upon reasonable request.
